# Tissues Harvested Using an Automated Surgical Approach Confirm Molecular Heterogeneity of Glioblastoma and Enhance Specimen's Translational Research Value

**DOI:** 10.3389/fonc.2019.01119

**Published:** 2019-10-23

**Authors:** Edie Zusman, Maxim Sidorov, Alexandria Ayala, Jimmin Chang, Eric Singer, Michelle Chen, Pierre-Yves Desprez, Sean McAllister, Nathan Salomonis, Kashish Chetal, Gautam Prasad, Tyler Kang, Joseph Mark, Lawrence Dickinson, Liliana Soroceanu

**Affiliations:** ^1^NorthBay Medical Center, Fairfield, CA, United States; ^2^California Pacific Medical Center (CPMC) Research Institute, San Francisco, CA, United States; ^3^Pacific Brain and Spine Medical Group, Eden Medical Center-Sutter Research, Castro Valley, CA, United States; ^4^Cincinnati Children's Hospital Medical Center (CCHMC) Biomedical Informatics, Cincinnati, OH, United States; ^5^NICO Corporation, Indianapolis, IN, United States

**Keywords:** glioblastoma, PDX, tumor heterogeneity, Myriad, RNA seq

## Abstract

Glioblastoma (GBM) is the most aggressive primary brain tumor in adults. Designing effective individualized therapies for GBM requires quality fresh tissue specimens, and a comprehensive molecular profile of this highly heterogenous neoplasm. Novel neuro-surgical approaches, such as the automated resection NICO Myriad™ system, are increasingly used by neurosurgeons to better reach the invasive front of tumors. However, no information exists on how harvesting GBM tissue using this approach may impact the translational research value of the sample. Here, we set out to characterize matched specimens from 15 patients, where one tissue sample was obtained using traditional tumor de-bulking (herein referred to as “en bloc” sample), and the other sample was obtained using the Myriad^TM^ System (herein referred to as “Myriad” sample). We investigated the fidelity of patient derived xenografts (PDXs) for each sample type to the corresponding human tissues and evaluated the added value of sequencing both samples for each patient. Matched en bloc and Myriad samples processed in parallel, were subjected to the following assays: cell viability, self-renewal, *in vivo* tumorigenicity using an orthotopic model of glioma, genomic sequencing, and pharmacological testing using PI3K-MTOR pathway inhibitors. Our results demonstrate that primary GBM cultures derived from matched specimens grew at similar rates (correlation coefficient *R* = 0.72), generated equivalent number of neurospheres, and had equivalent tumorigenic potential *in vivo* (mouse survival correlation coefficient *R* = 0.93). DNA Sequencing using the Illumina tumor panel amplicons revealed over 70% concordance in non-synonymous mutations between matched human GBM specimens. PDX genomic profiles were also highly concordant with the corresponding patient tissues (>70%). RNA sequencing of paired GBM samples revealed unique genomic variants and differential gene expression between the en bloc and Myriad specimens, with the former molecularly resembling the “tumor core” and the latter resembling the “invasive tumor front” signature. Functionally, we show that primary-derived GBM cells—obtained after fresh specimen's dissociation—are more effectively growth-inhibited by co-targeting non-overlapping mutations enriched in each sample type, suggesting that profiling both specimens more adequately capture the molecular heterogeneity of GBM and may enhance the design accuracy and efficacy of individualized therapies.

## Introduction

Glioblastoma (GBM), a grade IV glioma, according to World Health Organization (WHO) classification, is the most lethal primary glioma in adults. GBM has a prevalence of 26,000 cases, with a mortality rate of 15,000 cases yearly in the US, and an incidence of two to three per 100,000 adults per year (1). Currently approved therapies for GBM include surgery, radiation and Temozolomide (2). Surgical removal of the tumor via gross total resection is a critical determinant of patient outcome. Gross total resection of GBM increases the median survival rate by 200%, when compared to survival rates for patients subjected to a subtotal resection (3–5). While preserving as much un-involved brain tissue as possible, the surgeons aim to harvest sufficient tissue for molecular and pathological diagnosis, and for translational research (6). As such, patient derived cancer models, including patient derived xenografts (PDXs) and related cultures (PDX-C) have unique value in integrating the genomic data with drug sensitivity toward designing personalized care for GBM patients (7). Successful generation of PDX from freshly harvested GBM samples is an important measure of the specimen's translational value (6, 8).

The traditional GBM collection technique has been to remove a contiguous portion of the neoplasm from the surgical field with forceps and transport this *en bloc* specimen for processing. This method is less effective for collecting deep-seated, smaller lesions, and those located in eloquent brain regions, or for removing the “leading edge” of the neoplasm while preserving the adjacent normal white matter anatomy. The NICO Myriad^TM^ System (NICO Corporation, Indianapolis, IN) is a multi-functional, non-ablative, tissue resection device that uses a guillotine-like cutting aperture and variable suction to grasp and cut small targeted blocks of architecturally intact tissue (several cubic millimeters are harvested each minute). As the use of automated resection devices is showing improved outcome (especially for deep lesions), an important question arises regarding the translational research value of tissues harvested in this manner and how it compares molecularly with samples harvested en bloc. In this study, we set out to compare matched en bloc and Myriad-derived GBM samples obtained during tumor removal in 15 patients diagnosed with glioblastoma. For each pair, we generated primary cultures to assess cell viability, primary GBM neurosphere growth, as well as “mouse avatars” (patient derived xenografts, PDXs) to compare *in vivo* tumorigenicity and histopathological characteristics of matched xenografts. Next generation sequencing was used to interrogate mutations in genomic DNA using the Illumina tumor amplicon panel, both in matched patient samples and corresponding PDXs. Furthermore, RNA sequencing was used to identify genomic variants and analyze gene expression patterns in matched patients' samples.

### Ethics Statement

All patients included in the study were enrolled and consented for the study using an IRB approved non-treatment protocol (Sutter IRB# 25.125-2). All animal studies were pursued in accordance with CPMC approved IACUC protocols (protocol #15.08.03 and # 18.08.03).

## Methods

### Patient Selection and Preoperative Assessment

All patients participating in this study provided informed consent to have their tumor tissue collected for research purposes. All patients underwent magnetic resonance imaging (MRI) that was also used for intra-operative navigation to direct and assist with tumor resection.

### Operative Techniques and Tissue Collection Method

All tissues collected for the study were in excess of the sample required for pathological diagnosis (to guide patient treatment), per our IRB approved research protocol. Two methods of surgical access and tissue collection were used in this series including:

- Standard Craniotomy and en bloc collection followed by collection with the Myriad System.
- Minimally Invasive Parafascicular Surgery (MIPS) with en bloc collection followed by collection with the Myriad System.

For large, more superficial tumors, a standard craniotomy was performed, general practice guidelines were used to access the tumor through a large margin dural opening. Upon gaining access, an en bloc removal technique with tumor forceps was used to collect tissue in a traditional manner. At this time, sample for intra-operative pathological diagnosis was obtained. After GBM diagnosis confirmation by the pathologist, the collected specimen above mentioned is set-aside for tissue banking and transport in Hypothermosol solution (4°C). In each of these cases, a full time technician was present in the operating room during the full procedure dedicated to collecting and transporting the sample. Following en bloc resection of the tumor “core,” the NICO Myriad System was then utilized to remove additional tumor tissue bordering the uninvolved normal brain parenchyma; this sample was immediately refrigerated and placed in Hypothermosol solution and transported to the research lab together with the en bloc specimen, within 1.5 h.

For some deep-seated tumors, minimally-invasive parafascicular surgery (MIPS) was employed. This involved a small cranial flap and dural opening, and introduction of a 13.5 mm diameter, navigable, trans-sulcal tubular retractor (BrainPath®) at the base of a sulcus, staying parallel to DTI defined fiber tracts. Tumor was collected using a conventional en bloc technique with tumor forceps first. Then the adjacent tissue was removed with the Myriad System. Care was taken to assure that both specimens had identical MRI signal characteristics, suggesting that they shared morphologic and physiologic attributes. In both types of cases, substantial volume of tumor could be collected for both en bloc and Myriad-derived samples.

#### The Myriad System

The NICO Myriad™ System (NICO Corporation, Indianapolis, IN) is a multi-functional, non-ablative, targetable tissue resection tool used for tumor removal (9). It is used in conjunction with NICO's automated tissue preservation system (TPS) designed to standardize and automate the process of tissue collection, increase the volume of tissue collected for research, and potentially improve biological preservation through an intraoperative, automated process of refrigeration, and buffering of the collected tissue. The system also allows for regional targeting, resection, and annotation. Supplementary Figure 1 shows a diagram of the Myriad System as used in this study.

#### Primary GBM Cultures

Primary GBM cultures were generated as previously described (10). Cell viability was measured using automated cell counting (Countess II system) and performed in accordance with manufacturer instructions (ThermoFisher).

#### Next Generation Sequencing of Genomic DNA

Isolation of genomic DNA was performed using Qiagen kits after tissue disruption using ruptor disposable probes; DNA was quantified using PicoGreen (Thermo Fisher Scientific). A sequencing library targeting 212 amplicons in 48 genes was generated using the Illumina TruSeq Amplicon—Cancer Panel. Concordance between the original sample and its derivative was calculated as follows: Concordance: C = 100% ^*^ (*x*/*y*), where *x* = number of variants confirmed in both the en bloc and Myriad tissue and *y* = number of similar variants confirmed in en bloc tumor tissue or the Myriad tissue (11).

#### RNA Sequencing and Bioinformatics Data Analysis

RNA extraction from flash-frozen tissue samples was performed as previously described (12–14). Total RNA Extraction. RNA was processed from frozen tumor biopsies, following resuspension with Trizol (ThermoFisher Scientific). The total RNA was extracted by Direct-zol RNA Kit (Zymo Research) and the concentration was measured by Qubit RNA HS Assay Kit (ThermoFisher Scientific). RNA-Seq was performed from ~500 ng of total RNA processed using TruSeq polyA selection, at a target depth of 40 million paired-end, stranded reads on an Illumina 2500. Normalized gene expression data is available as Supplementary Table 2 in connection with this manuscript.

#### Gene Expression Analyses

For RNA-Seq analysis, gene expression values were obtained using the Kallisto algorithm in AltAnalyze version 2.1.1 from all FASTQ files, to obtain transcript per million (TPM) estimates. Differential gene expression was performed using an empirical Bayes moderated *t*-test, following FDR correction (*p* < 0.05). Additional gene set enrichment, hierarchical clustering and data visualizations were generated using AltAnalyze.

#### Identification of Cancer Genomic Variants

Genome variants were detected from the RNA-Seq data using the GATK RNA-Seq analysis workflow and annotated using the COSMIC database and Ensembl Variant Effect Predictor. Oncofusions were detected with the FusionCatcher pipeline. Additional variant and clinical annotations were obtained from the TCGA and TARGET consortiums. Variants were evaluated for statistical enrichment on biological Pathways (WikiPathways) using the GO-Elite algorithm in AltAnalyze and subsequently visualized through this software. Variant and oncofusion enrichment analyses were performed using a Chi-squared test (*p* < 0.05) aggregating variants at the gene level. Disease free and overall survival analyses were performed in R using the multivariate cox proportional hazard (coxph) tests for each splicing subtype. The R packages glmnet and coxph were used to test for other clinical covariates such as subtype/grade, cytogenetic abnormalities, relapse, induction failure, or secondary site of metastasis, while accounting for potential confounding variables such as age, gender, ethnicity, smoking, drug therapy, or subtype/grade. Enrichment analyses were assessed using Fisher's Exact Test *p*-values following FDR correction.

#### Taqman Validation of Gene Expression

Taqman Validation of Gene Expression for *REST* and *SEMA6B* was performed using primers and probes from Applied Biosystems according to the manufacturer's protocol as previously described by our group (15). Normal human brain RNA (Invitrogen, Cat# AM7962) was used as a positive control.

#### Pharmacological Studies and Cell Viability

Drugs were obtained from Selleckchem and master stocks were primarily made at half maximum solubility with DMSO. After drug treatment, cells were incubated for 72 h. CellTiter-Glo (Promega) was used to quantify cell viability/proliferation. Data from luminescence reads were used to calculate cell viability.

#### Glioma Neurosphere (Tumorsphere) Assays

Glioma Neurosphere (Tumorsphere) Assays were used to measure self-renewal potential of the glioma neurospheres, as previously described (10).

#### Immunofluorescence and Immunohistochemistry

Primary cultures were fixed using methanol (10 min, RT) and immunostained using the following primary antibodies (overnight incubation, 4C). Sox2 (1/1000, Epitomics), Tuj1 (Beta III Tubulin; 1/1000, Abcam). Nuclei were stained with DAPI or Propidium Iodide containing mounting medium from Vector Labs.

#### Intracranial Xenografts of Human GBMs

Intracranial Xenografts of Human GBMs were generated as previously published by our group (10, 12). Brains of euthanized mice were collected, fixed in formalin, paraffin embedded, and sectioned. Slides were stained with Hematoxlyin and Eosin, and then scanned using the Mirax MIDI whole slide high resolution scanning system.

## Results

### Patient Cohort

Fifteen patients who underwent surgical resection for glioblastoma standard of treatment were enrolled in the study. Table 1 lists patients' clinical information, including MGMT gene promoter methylation and IDH1 mutational status. Samples collected for this study were obtained using two different surgical approaches, as detailed below.

**Table 1:** Clinical annotation of GBM specimens included in this study.

**Case ID**	**Diagnosis**	**Age/Sex**	**MGMT methylation**	**IDH1/IDH2**
GBM 172	GBM	71 M	Not detected	N/D
GBM 177	GBM	66 F	Not detected	N/D
GBM 179	GBM	69 M	Not detected	N/D
GBM 180	GBM	54 F	Not detected	N/D
GBM 181	GBM	60 M	Not detected	N/D
GBM 183	Astrocytoma III	60 F	Not detected	R132H
GBM 190	GBM	77 F	Not detected	wild type
GBM 192	GBM	65 F	3.8%	Wild type
GBM 193	GBM	56 F	Not detected	Wild type
GBM 197	GBM	44 M	Not detected	Wild type
GBM 199	GBM	66 M	77%	Wild Type
GBM 203	GBM	65 M	70%	Wild type
GBM 208	GBM	82 M	Not detected	Wild type
GBM 213	GBM	49 F	Not detected	Wild type
GBM 215	GBM	72 M	Not detected	Wild type
GBM 216	GBM	68 M	Not detected	Wild type

### GBM Specimen Collection: Surgical Approaches

#### Standard Craniotomy

**Figure 1:**
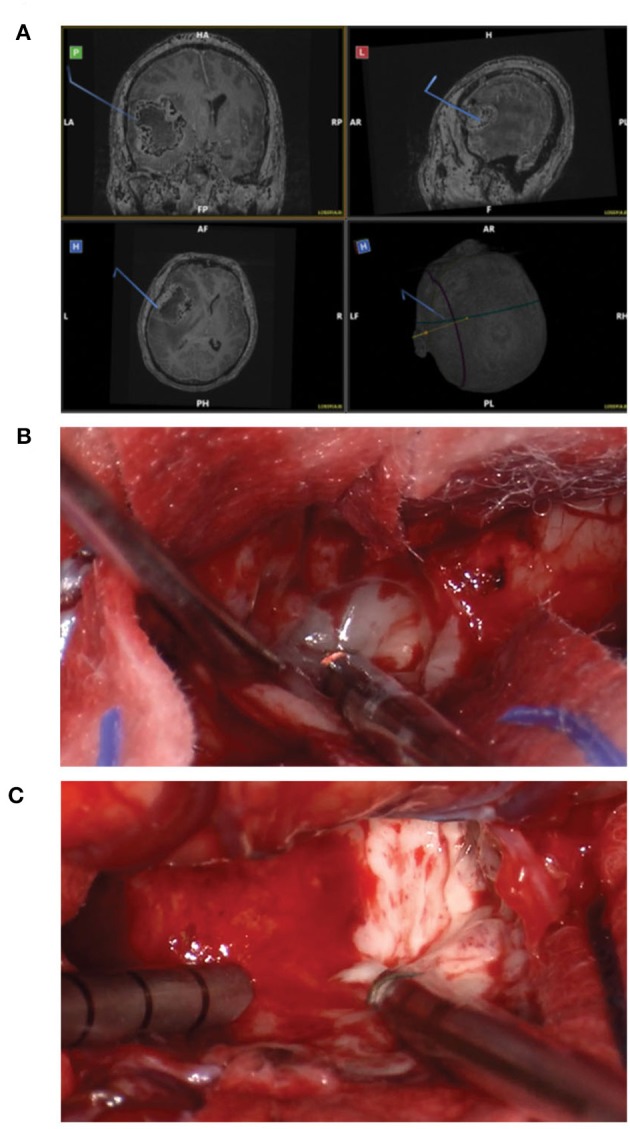
Standard craniotomy and the use of Myriad tool for surgical GBM removal. **(A)** Intraoperative navigation defining the area of tumor to be collected for the study. **(B)** Tumor cavity after *en bloc* resection with biopsy forceps has been completed and the collection with the Myriad System initiated. **(C)** Note the grayish blue color of the tumor, relative to the non-neoplastic edematous white matter.

Standard craniotomy with the option of using the Myriad System for tissue collection is the preferred approach whenever the tumor can be easily accessed. Figure 1 shows a representative case of one of the patients in our cohort undergoing a standard “open” craniotomy for resection of the tumor. Figure 1A shows the intraoperative navigation defining the area of tumor to be collected for the study. Figure 1B shows the tumor cavity after *en bloc* resection with biopsy forceps has been completed and the collection with the Myriad System (NICO Myriad™ and Automated Preservation System) initiated. Note the grayish blue color of the tumor, relative to the non-neoplastic edematous white matter appreciated after resection in Figure 1C.

#### Minimally Invasive Parafascicular Surgery (MIPS) With BrainPath

**Figure 2:**
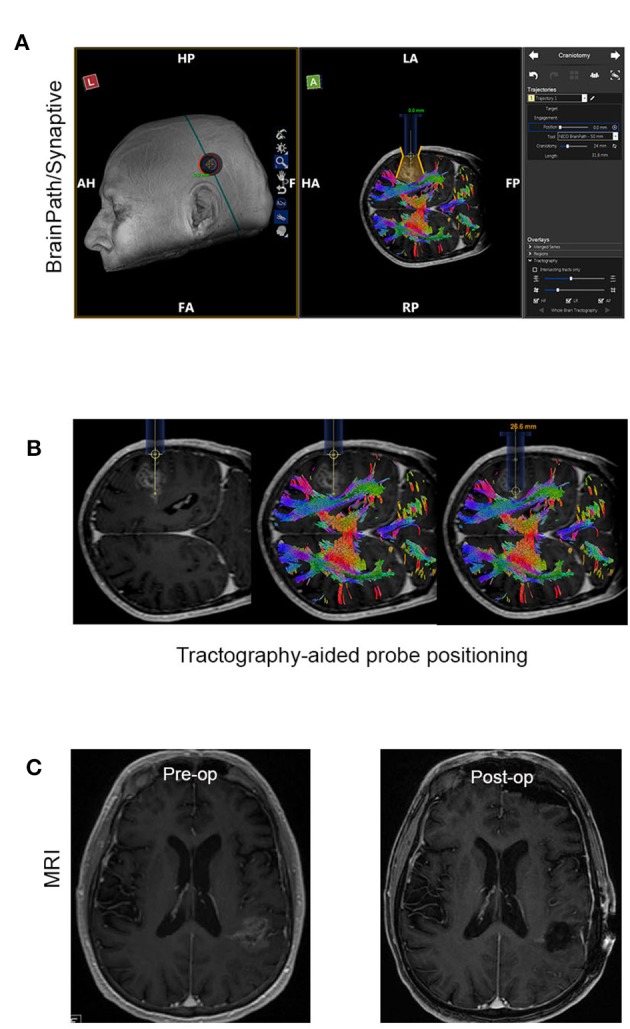
Representative case of a patient that had MIPS resection of the tumor. **(A)** Use of the planning software (Synaptive BrightMatter) to define a parafascicular trans-sulcal approach to the deep white matter neoplasm. **(B)** Intraoperative navigation with the target positioned at the deepest enhancing portion of the tumor (left panel). Tractography (shown in the middle panel) suggests that this area disease is within the corticospinal tract and this portion should not be resected. **(C)** Pre- and post op post gadolinium T1 MRI images with the small “tail” of residual enhancement in the corticospinal tract purposefully left behind.

Minimally Invasive Parafascicular Surgery (MIPS) with BrainPath was used to remove deep-seated tumors. This approach is also compatible with the use of the NICO Myriad System. Figure 2 is a representative case of a patient enrolled in our study who underwent MIPS for tumor resection. Figure 2A shows the use of Synaptive BrightMatter^®^ software to define a parafascicular trans-sulcal approach to the deep white matter neoplasm. The size of the craniotomy necessary to access the full dimension of the tumor through the 13.5 mm tubular retractor (NICO BrainPath®) is also shown on the planning image. Figure 2B shows the intraoperative navigation with the target positioned at the deepest enhancing portion of the tumor (left panel). However, it is evident on the tractography (middle panel) that this area of disease is within the corticospinal tract and this portion should not be resected, as it would cause right hemiparesis. The right panel in Figure 2B shows the operating channel in position for resection of the deepest portion of tumor collection that could be performed safely. Figure 2C shows the pre- and post op post gadolinium T1 MRI images with the small “tail” of residual enhancement in the corticospinal tract purposefully left behind. This patient had no neurologic deficit following the procedure.

After collection, both en bloc and Myriad specimens, were maintained according to this institution's protocol, in Hyothermosol solution (4°C) and transported within 1.5 h from surgery to the research lab for processing.

### *In vitro* Growth and Characterization of Primary-Derived GBM Neurospheres From Matched En Bloc and Myriad-Derived GBM Samples

**Figure 3:**
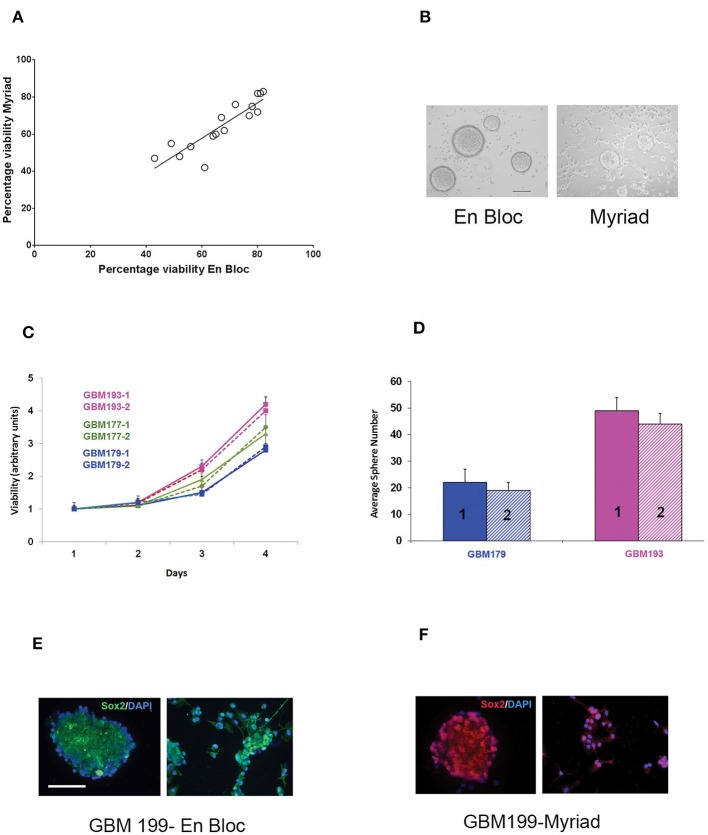
*In vitro* assessment of matched en-bloc and Myriad GBM samples. **(A)** Tumor cell viability was calculated based on counting 3 fields/sample. Initial cell viability values compared across 15 samples are shown. Correlation coefficient *R* = 0.72. **(B)** Representative photomicrographs of GBM tumor neurospheres from matched en bloc and Myriad–derived cultures. Bar = 200 μm. **(C)** Matched GBM primary cells from three patients (GBM 193, GBM177, GBM179) were cultured in complete growth media (96 well microplates, 5,000/well). Luminescence based viability readouts were obtained on days 2, 3, 4. Comparisons within each patient for matched cultures, *p* > 0.5 (student *t*-Test). Samples were run in triplicates; data is representative of two assays. **(D)** Tumorspheres growth in 24 well plates was quantified 7 days post initial culturing. 20,000 cells/well from matched GBM179 and GBM193 samples were allowed to form spheres in complete growth medium. Six replicates/condition were used and data is representative of 3 repeat assays; *p* = 0.4 Student *t*-Test. **(E,F)** Immunofluorescence analysis of primary GBM199 neurospheres. Sox2 and Tuj1 (right panels) are detected in both en bloc and Myriad-derived neurosphere cultures. *GBM 199-1* (en bloc): FITC- conjugated secondary antibodies and DAPI nuclear stain were used in **(E)**. *GBM 199-2* (Myriad) Cy3-conjugated secondary antibodies and PI nuclear stain were used in **(F)**. Bar: 150 μm.

Matched en bloc and Myriad-derived samples were processed in parallel to generate a single cell culture. Primary GBM cultures were established as previously described by our group (10). Initial cell viability and long term growth of neurosphere cultures from matched specimens were compared. Cell counts were obtained for 15 matched specimens following enzymatic and mechanical dissociation of fresh tissues, using the Countess II automated cell counter system. This approach is ideal for distinguishing between live and dead cells and also can accurately estimate cell viability of “clumpy” cultures which may result following GBM tissue dissociation. Median tumor cell viability was 56% for the en bloc specimen and 53% for the Myriad-derived specimen, with a correlation factor *R* = 0.72 between matched specimens (Figure 3A). GBM cultures were maintained as neurospheres for implantation and additional assays. Representative GBM-derived neurospheres obtained from matched patients' samples are shown in Figure 3B. Four day viability assays in three matched GBM samples demonstrate that similar growth rates characterize both the en bloc and Myriad-derived cultures, as shown in Figure 3C (*p* > 0.5 in all comparisons, Student *t*-Test). Neurosphere formation assays (a surrogate readout for tumor initiation potential) showed no difference between the en bloc and Myriad–derived samples. Figure 3D shows two examples of this assay performed using GBM 179 and GBM 193 samples (*p* = 0.8, Student *t*-Test). Next, we stained matched samples of primary patient GBM tumorspheres for markers associated with neural and glioma cancer stem cells which have previously shown to be enriched in primary glioblastoma samples (10, 15–17). Examples are shown in Figures 3E,F which display immunofluorescence-based detection of two neural stem markers—Sox2 and tubulin III (Tuj1) in matched specimens.

### *In vivo* Tumorigenicity and Histological Evaluation of Matched Patient and PDX Samples

**Figure 4:**
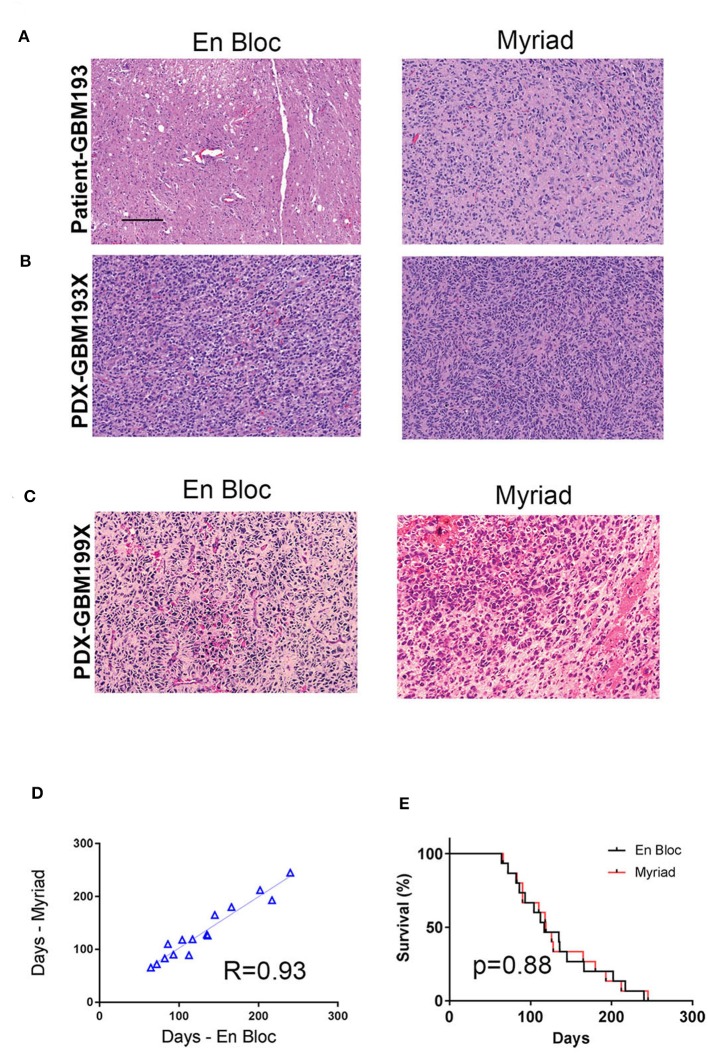
*In vivo* tumorigenicity assessment of matched en-bloc and Myriad GBM samples. **(A)** H&E staining of matched patient (GBM 193) samples are shown. **(B)** H&E for corresponding PDX–derived from GBM193. **(C)** H&E staining of PDX- derived from GBM199 en bloc (left panel) and Myriad (right panel) specimens; bar = 50 μm. **(D)** Survival correlation for mice intra-cranially implanted with 15 matched GBM samples (3,000,000 cells/mouse). Each sample was implanted in 2 mice (4 mice/patient). **(E)** Kaplan Meyer survival curves comparing survival between mice implanted with either en bloc or Myriad-derived tumor cells, for 15 patients. No significant difference in survival between the 2 cohorts is noted.

For *in vivo* tumorigenicity assessment, 300,000 cells were intracranially injected in two mice/specimen (four mice per patient) to generate PDXs. Mice bearing intracranial tumors were euthanized at the onset of neurological symptoms and animal survival was recorded for all samples. Intracranial xenografts are considered the best approach to test the ability of primary GBM cells to recapitulate the disease *in vivo*, and are characterized by high molecular and histological fidelity to patient's tumor (6). To validate the origin of tumors developed intracranially in nude mice, we performed hematoxylin and eosin (H&E) staining and evaluation of PDX tissue samples. Figure 4A shows an example of tissue sections from the *patient* (GBM 193, Figure 4A) and the corresponding *mouse PDX* (i.e., GBM193X, Figure 4B) stained using hematoxylin and eosin (H&E). GBM hallmarks, including high cellularity and areas of microvascular proliferation are noted. In another example, histological evaluation of GBM199-derived PDX tumor, is shown in Figure 4C. The H&E staining shows overall similar histological features between the matched xenografts, including areas of pseudo-palisading, another hallmark feature of GBM (18).

*In vivo* tumor take for all 15 paired samples (60 mice) was 100%. The time required for intracranial development of tumors (measured from time of implantation until neurological symptoms were present) was proportional to the tumor grade, as previously described (19). *In vivo* tumorigenicity correlation between matched en bloc and Myriad-derived samples was *high, R* = 0.93 (Figure 4D). Survival of mice implanted with either en bloc or Myriad-derived GBM cells (300,000/mouse, intra-cranially) is shown in Figure 4E for all 15 patients. Kaplan Mayer survival analysis shows no significant difference in survival between the two cohorts (Log-rank Mantel-Cox test, *p* = 0.88).

### Genomic Characterization of Matched GBM DNA Samples

Next generation sequencing using MiSeq tumor panel was performed on genomic DNA extracted from 13 matched patient samples. Additionally, five distinct PDX samples were sequenced to interrogate fidelity of the PDX model. Genomic DNA sequencing was performed using the AmpliSeq for Illumina Cancer Hotspot Panel v2. This is a targeted assay for identification of somatic mutations across the hotspot regions of 50 genes with known associations to cancer, as identified in the Catalog of Somatic Mutations in Cancer (COSMIC) database. Supplementary Table 1 shows allele frequency information for the non-synonymus mutations identified in 7 genes previously shown to be involved in GBM pathogenesis (20, 21).The numbers shown in each cell represent variant allele frequencies (VAF). VAF in this case refers to the fraction of sequencing reads overlapping a genomic coordinate that support the mutant allele. The values shown correspond to the most deleterious variant identified within each gene. We calculated the percentage concordance between en bloc and Myriad samples from the same patient. Our data shows between 71 and 100% concordance in VAF between matched GBM samples from the same patient. This high degree of concordance is not surprising, given that the Illumina tumor amplicon panel used here includes a subset of genomic alterations frequently reported in GBM. Concordance between each PDX and its corresponding patient tumor was calculated to be 70% or higher.

Taken together, the data so far supports the notion that the use of the Myriad tool for GBM resection results in highly viable and tumorigenic tissue samples, similar to those obtained using the traditional, en bloc approach.

### RNA Sequencing and Differential Gene Expression in Paired Samples

**Figure 5:**
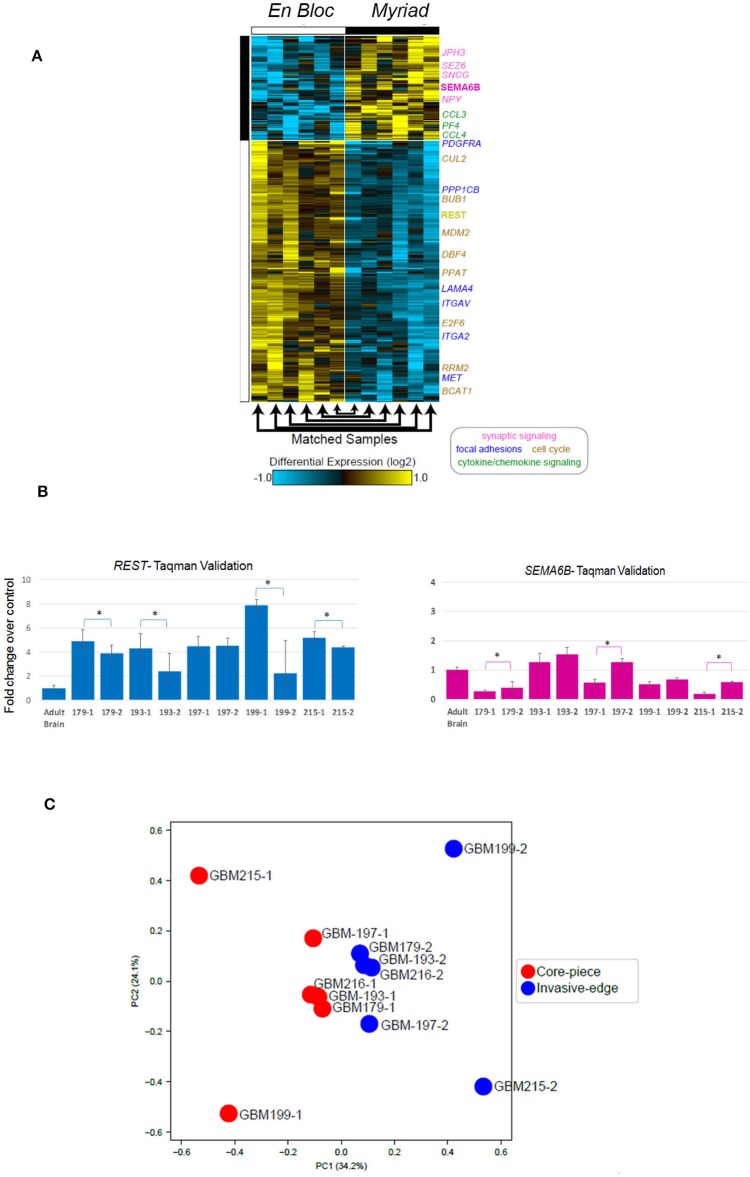
Differentially expressed genes in paired GBM specimens. **(A)** Heatmap displaying genes differentially expressed in the en bloc and Myriad specimens across 12 GBM specimens. Genes with a fold change > 1.5 and FDR adjusted, e Bayes moderated *t*-test *p* < 0.05. Yellow indicates up-regulation and blue, down-regulation, relative to the median expression across all samples. Highlighted genes are indicated to the right of the heatmap, colored according to their indicated biological process (i.e., synaptic signaling, cell cycle, etc). **(B)** Taqman validation in 5 matched GBM samples for REST (left panel) and SEMA3B (right panel). *p < 0.02 Student *T*-test. **(C)** Principal component analysis of all expressed genes (TPM > 1), followed intra-patient normalization.

We used RNA Seq to interrogate gene expression in matched GBM specimens, in order to test the hypothesis that tissue samples from different anatomical regions of the tumor exhibit distinct molecular profiles, corresponding to multiple cancer driving signaling pathways (21). Figure 5A illustrates hierarchical clustering of differentially expressed genes in 12 specimens (6 matched en bloc and Myriad samples). Highlighted genes are shown to the right of the heatmap, colored according to their indicated biological process (e.g., cell cycle in yellow font, cytokine signaling in green font, synaptic signaling in purple font). Note that genes involved in cell cycle and focal adhesion regulation were enriched in the en bloc specimen, while genes regulating chemokine and synaptic signaling were upregulated in the Myriad sample, respectively. Next, we performed Taqman validation measuring expression levels for the cell cycle related gene *REST*, and a gene associated with synaptic signaling -*SEMA6B*, in 10 matched en bloc and myriad RNA samples. Significant differences in expression levels for each of these genes are noted in individual sample pairs (Figure 5B).

### GBM Regional Heterogeneity Is Revealed by Analysis of Both En Bloc and Myriad Samples

*Principal component analysis* followed by intra-patient normalization, of all expressed genes demonstrates that the en bloc samples from all six patients cluster *together* (red full circles) and *away* from the matched Myriad samples for the same patients (blue full circles; Figure 5C). This suggests distinct biological pathways are enriched in each sample type, across all analyzed patients; these pathways may play complementary roles in GBM progression. GBM regional heterogeneity has been extensively documented (22). In a recent study, single cell RNA Seq of neoplastic cells, showed differential gene expression between cells retrieved from the tumor core and those collected from the invasive/migrating tumor edge (23). This study identified differentially expressed genes in these two tumor regions, associated with distinct signaling pathways. As such, the tumor-core enriched gene ontology (GO) pathways were cell proliferation, hypoxia-induced pathways, and cancer stem cells-related pathways, while GO pathways associated with the tumor edge and immune cell infiltrates included chemokine and chemokine receptors, cytokines, nervous system development, and cell migration related pathways (21). Using the open source software and data base available in conjunction with this publication (http://www.gbmseq.org) we analyzed RNA Seq data from the 6 matched GBM tissues (total 12 samples) and identified several genes overexpressed in the *en bloc* samples corresponding to the *tumor core* (as defined by single cell RNA expression profiling), while a number of genes overexpressed in the *Myriad samples* were enriched in the *invasive edge* of the tumor as defined by single cell RNA expression profiling. Relative abundance levels and corresponding tumor region for six genes are shown in Supplementary Figure 2. Statistically enriched Gene Ontology terms associated with up- or downregulated genes in the en bloc (tumor core) vs. Myriad (tumor edge) samples are shown in Supplementary Figure 3. Enrichment Fisher Exact was *p* < 0.001 for all pathways (GO-Elite). Taken together, these data suggest that molecular sequencing of both samples provides a comprehensive molecular tumor fingerprint in GBM, which is also actionable information for guiding individualized therapy.

### Genomic Variants and Wiki-Pathway Analyses in Paired Samples

The 12 matched samples analyzed by RNA Seq were further interrogated to identify specific genomic variants for each patient. These data were analyzed using the AltAnalyze and WikiPathways platforms to identify signaling pathways specifically activated within each sample, based on predicted activating mutations. Examples of WikiPathway analyses for three matched GBM samples are shown in Supplementary Figures 4–6, where mutated genes are shown in red. Overall consistency in pathway activation is noted between matched samples. However, a subset of genomic alterations are identified in one specimen, but not the other (blue open circles). For example, an *ATM* mutation is only present in GBM 179-1 (en bloc sample), but not in GBM179-2 (Myriad; Supplementary Figure 4). *EGFR/ERBB2* alterations were identified only in the GBM199-2 (Myriad) but not in the GBM 199-1 (en bloc) sample (Supplementary Figure 5), while a *CDK4* mutation is uniquely present in the GBM216-1 (en bloc) but not in GBM216-2 (Myriad; Supplementary Figure 6). While mechanistic studies are required to fully investigate the significance of these sample-specific genomic alterations, it is clear that gathering sequencing information from both specimens results in a more accurate and complete molecular characterization of the tumor.

**Figure 6:**
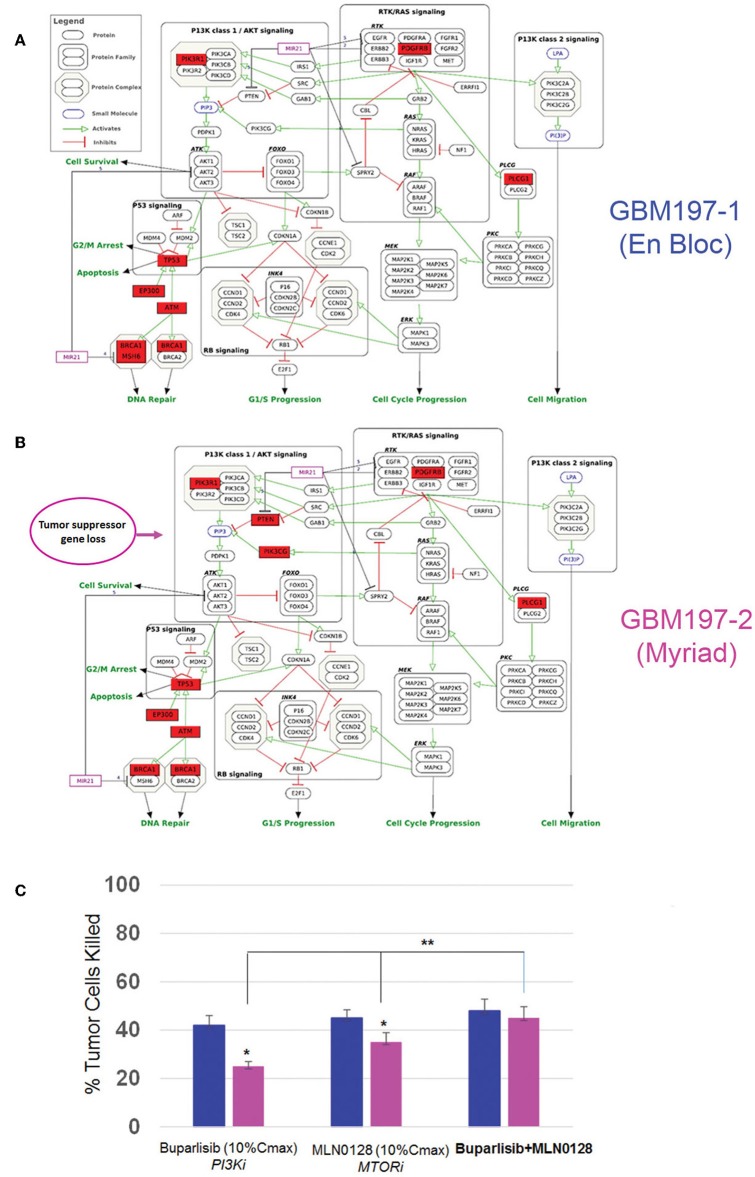
Genomic and drug sensitivity testing in matched en bloc and Myriad GBM samples. **(A,B)** Wikipathways analysis for en bloc (GBM197-1, **A**) and Myriad (GBM197-2, **B**) samples. Arrow indicates the presence of a PTEN alteration in the Myriad specimen only (7B). **(C)** Cell viability data for GBM197-1 (blue bars) and GBM197-2 (pink bars) treated with a PI3K inhibitor (Buparlisib, 0.2 μM) and an MTOR inhibitor (MLN0128, 0.1 μM) 72h. Dug concentrations used in this assay represent 10% Cmax measured in the plasma from patients. *p = 0.04, comparing single drug treatment and **p = 0.01 comparing combination drug treatment with single drug treatment in the Myriad –derived specimen. Student *t*-Test.

To test the functional significance of these genomic findings, we generated primary cultures from matched GBM197-1 (en bloc) and GBM197-2 (Myriad) samples and subjected the cells to pharmacological testing. Pathway analyses for GBM197-1 (en bloc) and GBM 197-2 (Myriad) are shown in Figures 6A,B. Note that a critical tumor suppressor gene (*PTEN)* is altered in GBM197-2 only. Drug sensitivity testing was performed using Buparlisib which inhibits the PI3K pathway and MLN0128, an investigational MTOR inhibitor. As shown in Figure 6C, the 197-2 derived cells appear more resistant to treatment with either inhibitor compared to 197-1 cells, which is consistent with enhanced activation of the PI3K pathway driven by alterations in *PTEN* uniquely present in this sample. Combining both drugs was significantly more efficacious in inhibiting the growth of GBM197-2 cells.

Taken together, these data suggest that capturing the genomic heterogeneity of GBM by closely analyzing both en bloc and the Myriad-derived specimens may be required for designing effective personalized targeted or immunotherapy approaches, including neoantigen-based vaccine therapy for GBM patients (24).

## Discussion

Clinical trials for GBM are investigating efficacy of individualized targeted therapy based on genomic profiling as well immunotherapy options. The availability of high quality, viable tumor tissue is critical for the aforementioned efforts. In this study, we demonstrate that an automatic tissue collection and preservation strategy used to harvest GBM specimens proved equal to the traditional “en bloc” tumor resection in terms of providing viable samples for translational research. Our data attest to the viability and tumorigenicity of both Myriad (NICO Myriad™) and en bloc-derived specimens as shown by cell viability and tumor sphere growth assays in culture. In addition we document expression of molecular markers associated with self-renewal in primary GBM cultures derived from both types of specimens. The ability of tumor cells to recapitulate the disease in an immunocompromised mouse constitutes critical evidence of tissue viability. Our data shows that primary GBM cells from 15 matched specimens uniformly generated intracranial tumors in nude mice. Histo-pathologically, PDXs derived from each specimen recapitulated hallmark features of glioblastoma (7). Genomic sequencing confirmed the presence of “hot spots” in both specimens, with a high degree of concordance. Interestingly, RNA sequencing and bioinformatics analyses revealed salient gene expression differences between the two specimens and identified sample-specific genomic variants. In terms of gene expression, all en bloc samples clustered together and away from the Myriad-samples, as demonstrated by the principal component analysis. Furthermore, the gene expression signature enriched in the en bloc specimen was characteristic of the “tumor core,” while the gene expression profile of the Myriad specimens corresponded to the “invasive tumor front” (23). In a recent study by Sottoriva et al., intra-tumor heterogeneity was assessed using surgical multisampling from same patient (similar to our approach) and various glioblastoma subtypes were identified within the same tumor (25). Similarly, our study demonstrates the presence of distinct tumor-promoting signaling pathways in the two matched GBM specimens, driven by non-overlapping genomic variants. These results are in agreement with previous studies who documented GBM gene expression heterogeneity at the single cell level. The study by Patel et al. (26) demonstrated the presence of diverse transcriptional programs related to oncogenic signaling, proliferation, and immune response in glioblastoma and their implication for disease progression. Regional molecular heterogeneity may have important consequences for designing combinatorial therapies for GBM. The PTEN-PI3K-MTOR pathway is altered in over 70% of GBMs (27, 28). Preliminary results from clinical trials in a subset of GBMs suggested that inhibition of any of the pathway's nodes alone was not efficacious, because of paradoxical pathway activation (29, 30). Concordantly, our data show that combined inhibition of PI3K and MTOR was significantly more efficacious in inhibiting GBM197 growth than either drug alone. Together, these results suggest that identification of molecular “fingerprints” in GBM samples by sequencing both the en bloc and the Myriad specimens, provides more comprehensive information about disease-driving pathways. The ability to annotate anatomical features with distinct genomic makers can be used for drug target validation and thus aid in designing of combinatorial therapies for patients with GBM (31).

In summary, this novel method of GBM collection and preservation was at least equivalent to or an improvement on traditional tissue harvesting, and permitted more controlled tissue removal at the tumor edges. We also found that en bloc tissues, usually obtained from the tumor “core” cluster *together* and display distinctly different RNA signatures than the Myriad derived tissues, usually obtained from the tumor edge. These data highlight the important issue of GBM heterogeneity as it relates to distinct regions within the tumor, harvested using specific surgical approaches (suitable for each location). Our results further support the need to harvest and sequence multiple specimens for each GBM, in order to capture the genomic diversity within each patient's tumor and improve the design of molecularly-directed therapeutics.

## Data Availability Statement

This manuscript contains previously unpublished data. The name of the repository and accession number(s) are not available. Normalized gene expression data for all specimens included in this manuscript is available as Supplementary Table 2.

## Ethics Statement

The studies involving human participants were reviewed and approved by Sutter IRB. The patients/participants provided their written informed consent to participate in this study. The animal study was reviewed and approved by IACUC, CPMC Research Institute.

## Author Contributions

LS, EZ, LD, and JM conceived and designed the study. MS, AA, JC, ES, MC, P-YD, SM, and LS collected samples and performed experiments. EZ, LD, GP, and TK contributed patient samples and annotation. NS and KC performed bioinformatics analyses. EZ, LS, LD, and JM analyzed the data. LS, LD, and JM wrote the manuscript.

### Conflict of Interest

The authors declare that this study received funding from Nico Corporation. The funder had the following involvement with the study: advised on tissue collection using the Myriad tool (as described under section “Methods”). JM was employed by the Nico Corporation. The remaining authors declare that the research was conducted in the absence of any commercial or financial relationships that could be construed as a potential conflict of interest.

## Supplementary Material

Supplementary Figure 1 **(A)** Minimally Invasive Parafascicular Surgery (MIPS) Access Device, the BrainPath® (NICO Corporation, Indianapolis, IN) of various lengths and diameters. **(B,C)** The NICO Myriad™ System Components: **(B)** Side mouth aperture, filter element and specimen collector of the Myriad System are indicated. **(C)** Tissue Preservation System (TPS) components are shown, including the Tissue Filter Assembly, as well as Specimen Preserver and Chiller.

Supplementary Figure 2 Genes enriched in the en bloc samples are enriched in the tumor core and genes overexpressed in Myriad samples are enriched in the invasive tumor front. Shown here are *LAMA4, REST, BUB1*, and *MDM2* enriched in en bloc samples, and *CCL3* and *SEZ6* enriched in the Myriad samples. Gene names are color coded based on the pathways associated with (see also Figure 6).

Supplementary Figure 3 Statistically enriched Gene Ontology terms associated with up or downregulated genes with enrichment Fisher Exact *p* < 0.01 (GO-Elite). Inferred interaction network of focal adhesion genes (direct interactions), Cytokine-cytokine receptor interactions (indirect interactions), and synaptic signaling genes (indirect interactions) were generated based on protein-protein interactions from pathway databases (WikiPathways, KEGG, BioGRID) using the software NetPerspective in AltAnalyze.

Supplementary Figure 4 WikiPathway analyses in matched GBM 179 samples. Pathways implicated in GBM progression (e.g., PI3K-AKT, RTK/RAS, cell cycle progression, DNA repair) are shown for matched en bloc and Myriad samples from GBM179. Mutated genes are shown in red. Blue circles delineate non-overlapping genomic alterations identified in the two matched specimens for each patient.

Supplementary Figure 5 WikiPathway analyses in matched GBM 199 samples. Pathways implicated in GBM progression (e.g., PI3K-AKT, RTK/RAS, cell cycle progression, DNA repair) are shown for matched en bloc and Myriad samples from GBM199. Mutated genes are shown in red. Blue circles delineate non-overlapping genomic alterations identified in the two matched specimens for each patient.

Supplementary Figure 6 WikiPathway analyses in matched GBM 216 samples. Pathways implicated in GBM progression (e.g., PI3K-AKT, RTK/RAS, cell cycle progression, DNA repair) are shown for matched en bloc and Myriad samples from GBM216. Mutated genes are shown in red. Blue circles delineate non-overlapping genomic alterations identified in the two matched specimens for each patient.

Supplementary Table 1 Identification of genomic “hot spots” in matched GBM samples. **(A,B)** Matched samples and corresponding PDX (when available) from each patients are shown in a different color in the chart (total of 13 patients). En bloc sample is designated−1 and Myriad samples is designated−2. GBM179-1X corresponds to the PDX generated from GBM179-1; GBM179-2X designates the PDX generated from GBM 179-2 tissue. Genomic DNA from matched samples was extracted from fresh frozen tissues and sequencing was performed using the MiSeq machine in conjunction with the Illumina tumor amplicon panel. The numbers shown in each cell represent allele frequencies (VAF). VAF between 0.1 and 1 are within the sequencer's detection limit. Concordance in VAF between matched specimens and with the corresponding PDX are also shown.

Supplementary Table 2 Normalized gene expression values for 6 matched GBM patient samples (12 total) obtained by RNA Sequencing.
