# Supplementary figures and images for: Tissues Harvested Using an Automated Surgical Approach Confirm Molecular Heterogeneity of Glioblastoma and Enhance Specimen's Translational Research Value
Source: Front Oncol. 2019 Oct 23;9:1119. doi: 10.3389/fonc.2019.01119 (PMC6843001; doi:10.3389/fonc.2019.01119)

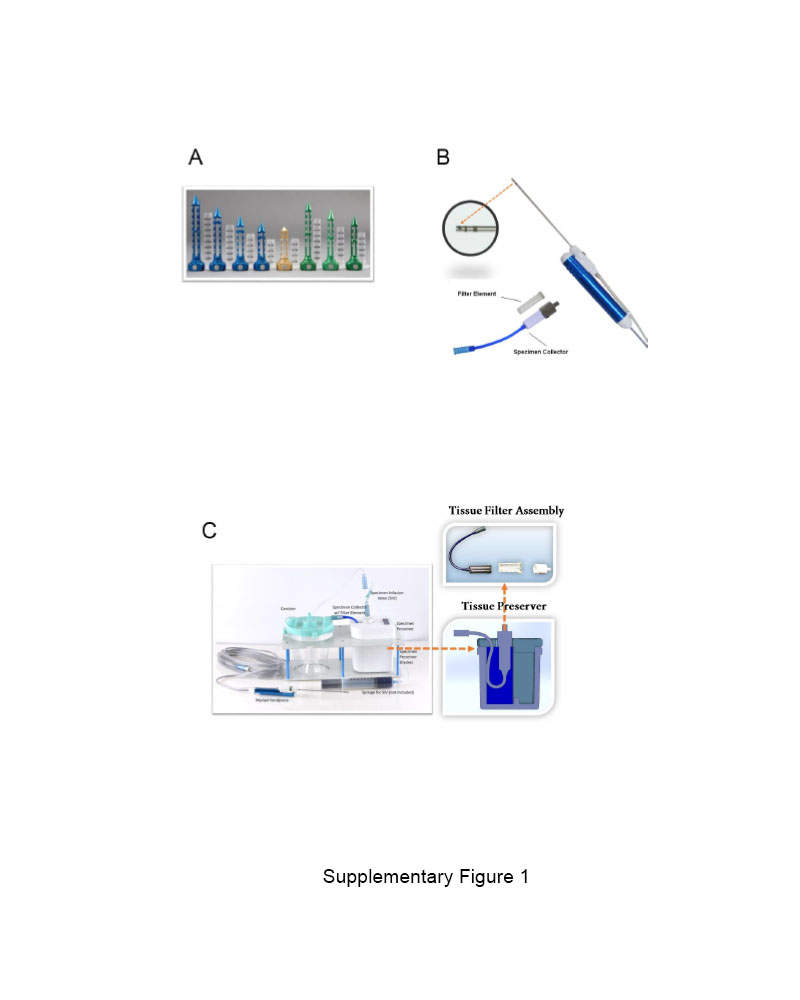

Supplement: Supplementary Figure 1 — (A) Minimally Invasive Parafascicular Surgery (MIPS) Access Device, the BrainPath® (NICO Corporation, Indianapolis, IN) of various lengths and diameters. (B,C) The NICO Myriad™ System Components: (B) Side mouth aperture, filter element and specimen collector of the Myriad System are indicated. (C) Tissue Preservation System (TPS) components are shown, including the Tissue Filter Assembly, as well as Specimen Preserver and Chiller. [file Image_1.JPEG]

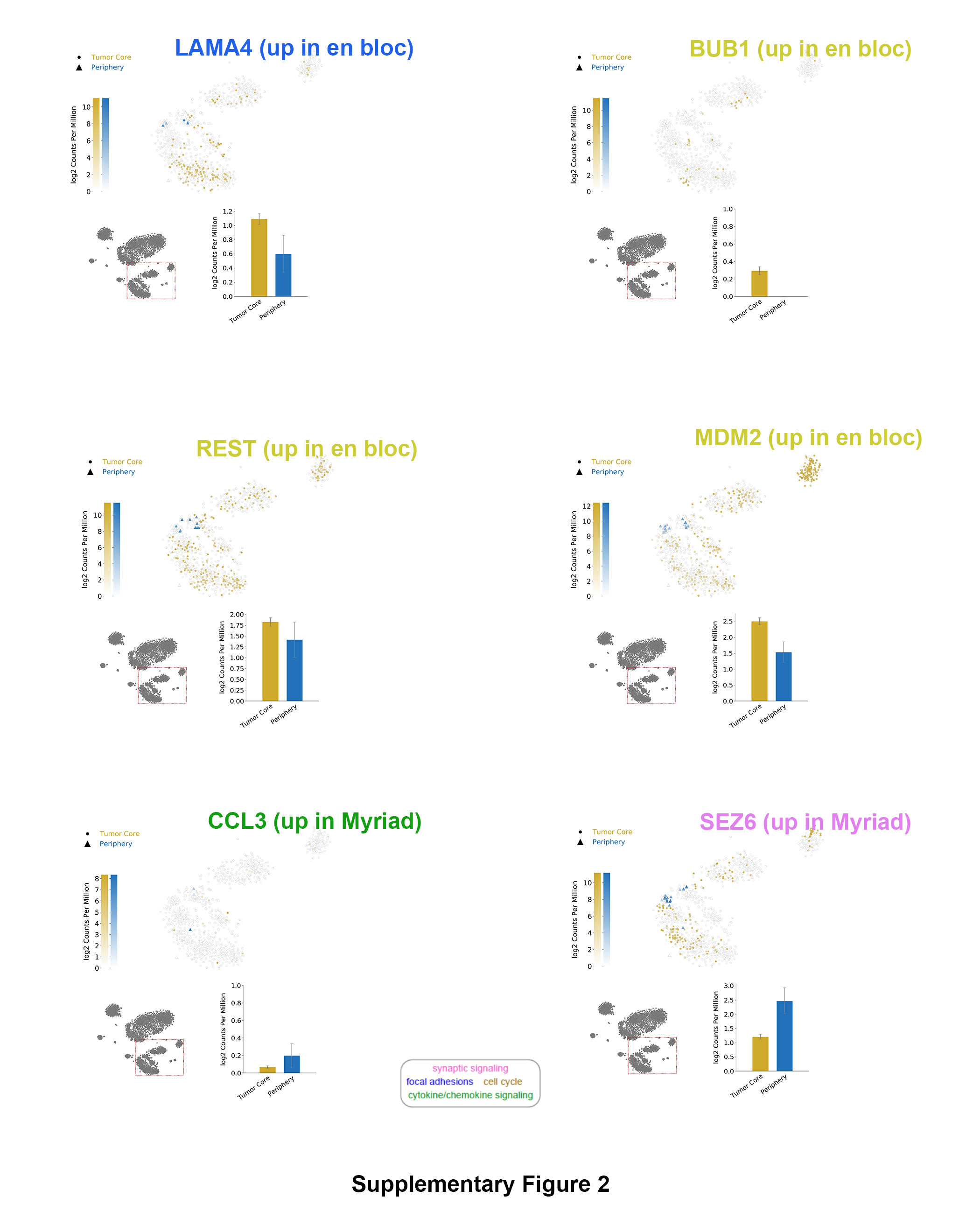

Supplement: Supplementary Figure 2 — Genes enriched in the en bloc samples are enriched in the tumor core and genes overexpressed in Myriad samples are enriched in the invasive tumor front. Shown here are LAMA4, REST, BUB1, and MDM2 enriched in en bloc samples, and CCL3 and SEZ6 enriched in the Myriad samples. Gene names are color coded based on the pathways associated with (see also Figure 6). [file Image_2.JPEG]

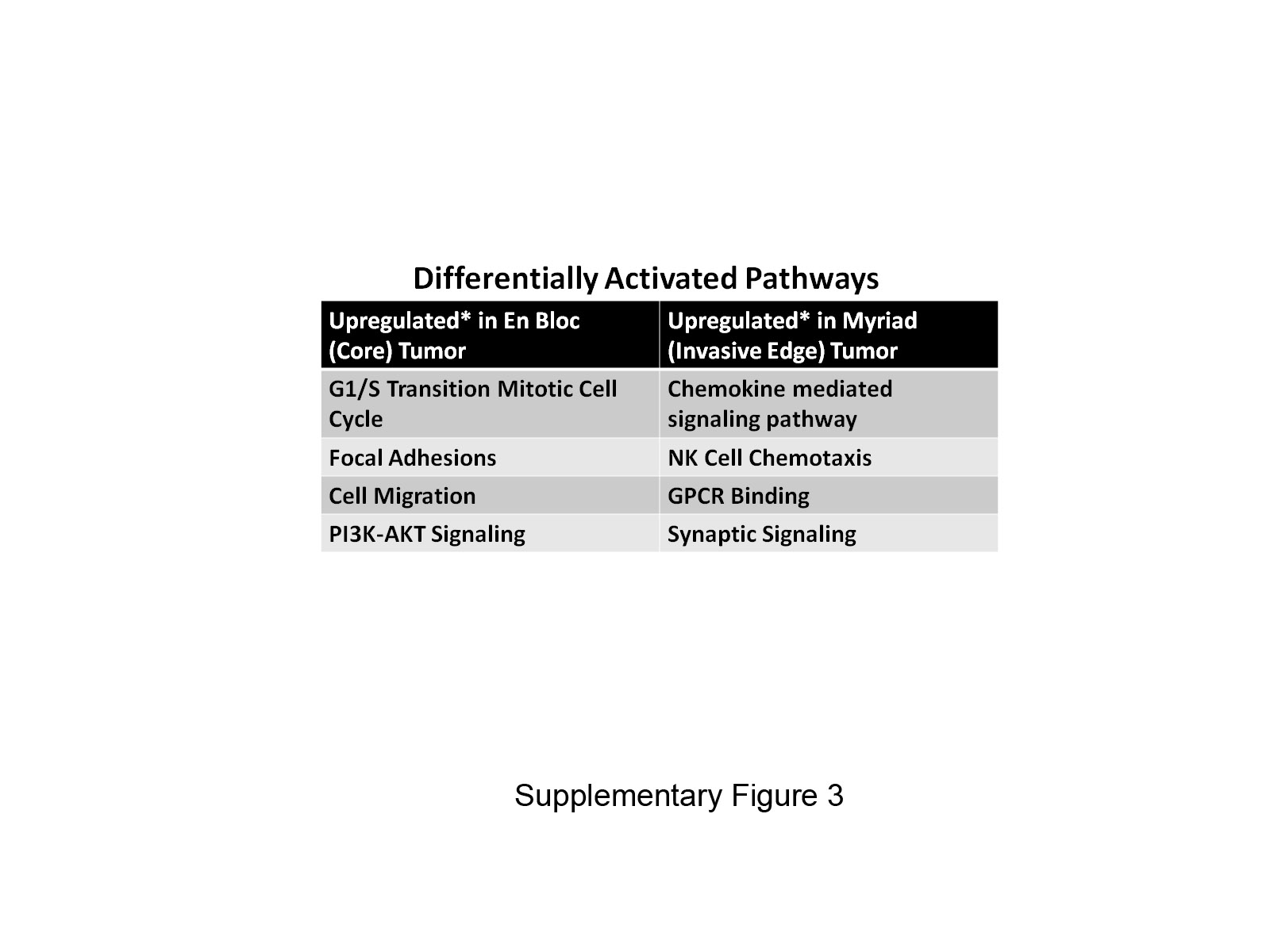

Supplement: Supplementary Figure 3 — Statistically enriched Gene Ontology terms associated with up or downregulated genes with enrichment Fisher Exact p < 0.01 (GO-Elite). Inferred interaction network of focal adhesion genes (direct interactions), Cytokine-cytokine receptor interactions (indirect interactions), and synaptic signaling genes (indirect interactions) were generated based on protein-protein interactions from pathway databases (WikiPathways, KEGG, BioGRID) using the software NetPerspective in AltAnalyze. [file Image_3.JPEG]

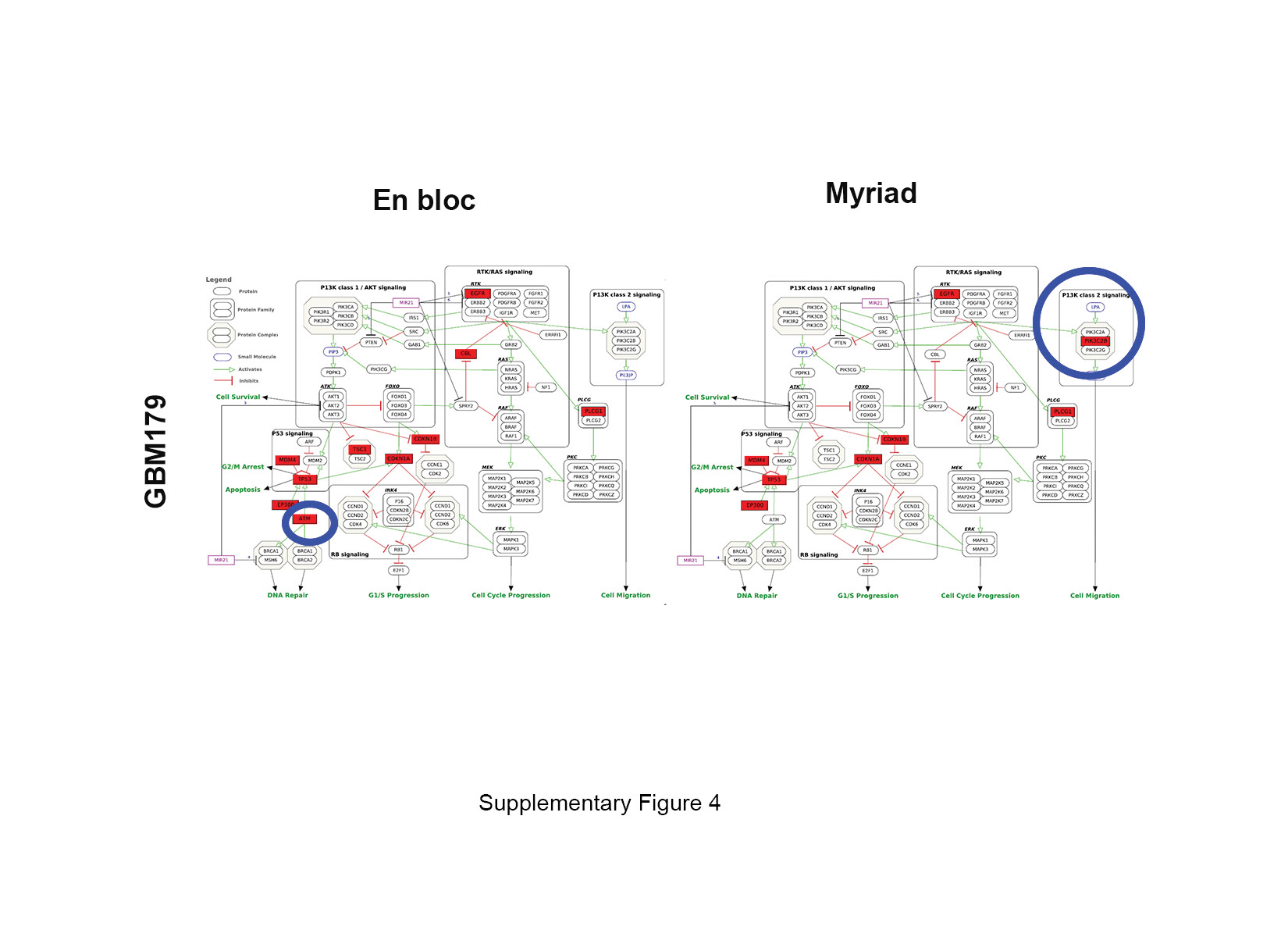

Supplement: Supplementary Figure 4 — WikiPathway analyses in matched GBM 179 samples. Pathways implicated in GBM progression (e.g., PI3K-AKT, RTK/RAS, cell cycle progression, DNA repair) are shown for matched en bloc and Myriad samples from GBM179. Mutated genes are shown in red. Blue circles delineate non-overlapping genomic alterations identified in the two matched specimens for each patient. [file Image_4.JPEG]

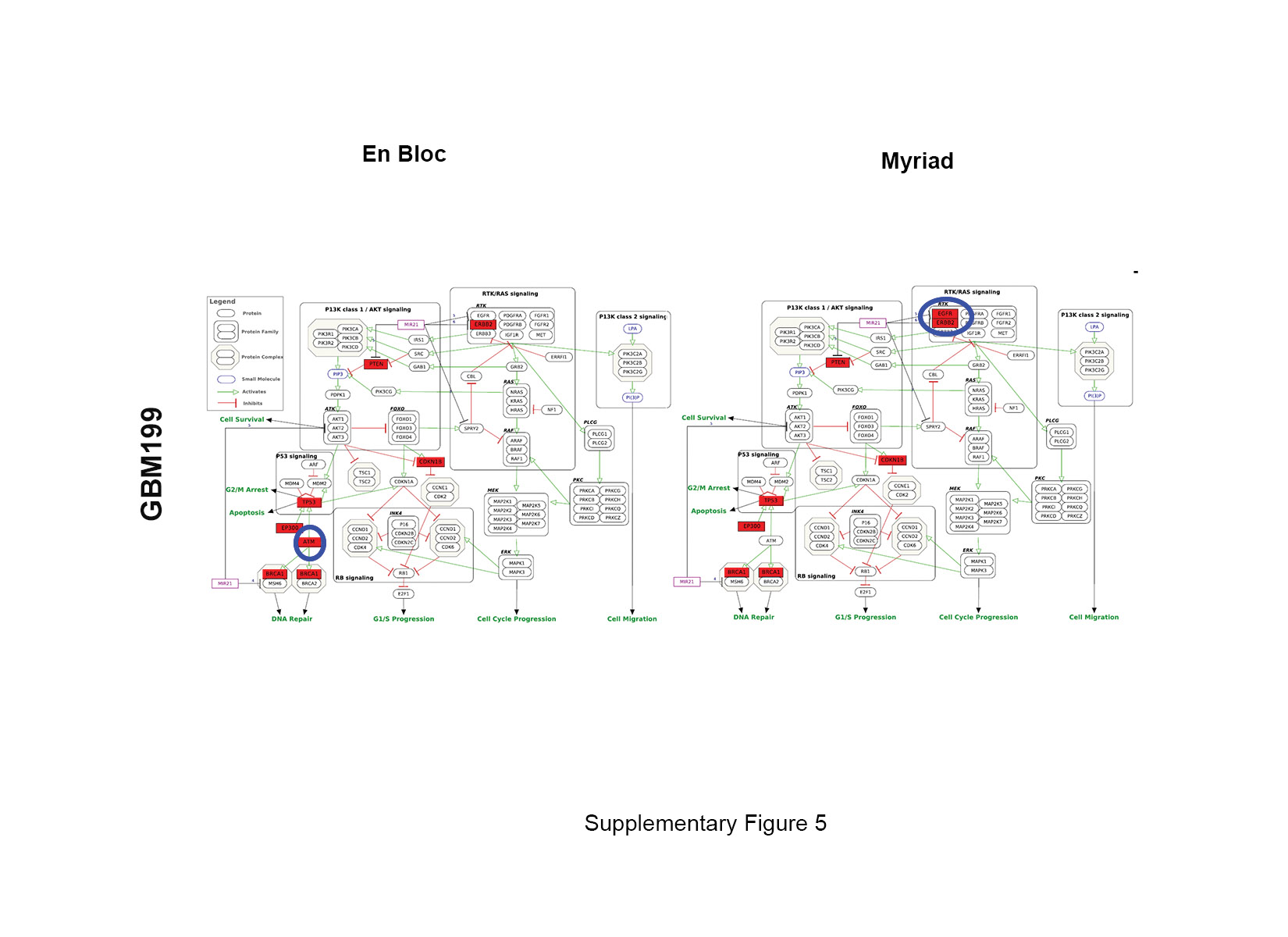

Supplement: Supplementary Figure 5 — WikiPathway analyses in matched GBM 199 samples. Pathways implicated in GBM progression (e.g., PI3K-AKT, RTK/RAS, cell cycle progression, DNA repair) are shown for matched en bloc and Myriad samples from GBM199. Mutated genes are shown in red. Blue circles delineate non-overlapping genomic alterations identified in the two matched specimens for each patient. [file Image_5.JPEG]

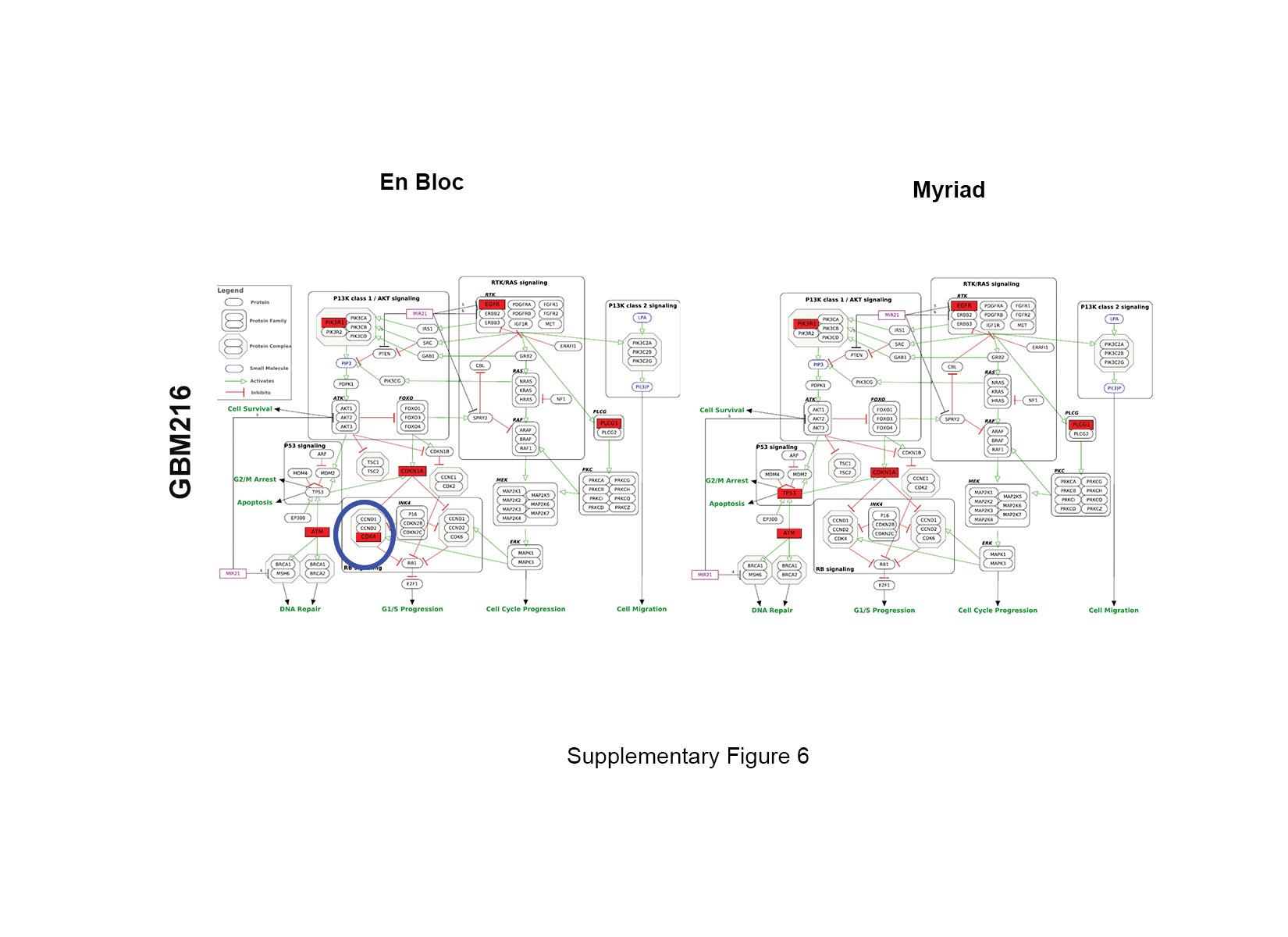

Supplement: Supplementary Figure 6 — WikiPathway analyses in matched GBM 216 samples. Pathways implicated in GBM progression (e.g., PI3K-AKT, RTK/RAS, cell cycle progression, DNA repair) are shown for matched en bloc and Myriad samples from GBM216. Mutated genes are shown in red. Blue circles delineate non-overlapping genomic alterations identified in the two matched specimens for each patient. [file Image_6.JPEG]
